# The new paradox of women’s health research

**DOI:** 10.7554/eLife.113335

**Published:** 2026-10-23

**Authors:** Ursula Debarnot, Alexandre Fouré

**Affiliations:** 1 Inter-University Laboratory of Human Movement (LIBM), Université Lyon 1 Lyon France

**Keywords:** women in science, gender, sex differences, inclusivity, women's health, None

## Abstract

Women have long been underrepresented in biomedical research, prompting efforts to make research more sex inclusive. These advances are essential, but they can create an unintended tension when rigorous inclusion is interpreted as requiring extensive hormonal or reproductive characterization, even when such detail is unnecessary for the question being asked. We examine this paradox, in which standards intended to strengthen research on women may inadvertently make some studies involving females harder to pursue. We argue that characterization should match the claim a study is designed to support, and that funders, institutions and journals must provide the resources needed to make such rigor feasible. Methodological progress should ultimately broaden, not narrow, the evidence base needed to improve women’s health.

## From inclusion to characterization

It all started with designing a study to examine how sleep influences recovery from exercise-induced muscle damage in women and men. As the protocol developed, our respective fields brought different methodological considerations. Evidence that ovarian steroid profiles may influence muscle damage and recovery raised clear mechanistic issues, whereas possible sex-related differences in sleep physiology remained a more exploratory aspect of the project (Jomard and Gondin, 2023; Walsh et al., 2026). Bringing these perspectives into a single protocol made questions of menstrual cycle phase, hormonal verification, scheduling and interpretation increasingly difficult to separate from the study design itself. We concluded that including women would require a level of biological characterization and logistical organization beyond our available resources, a judgement reinforced by the methodological standards we ourselves might apply when reviewing similar research. We therefore restricted recruitment to men.

This decision echoed a longstanding asymmetry in biomedical research. Women have been historically excluded or underrepresented, while evidence generated largely from male participants often served as the default; reproductive cycling and presumed biological variability were among the reasons used to justify this imbalance (Ahmed et al., 2026; Mauvais-Jarvis et al., 2020). Policy changes and scientific initiatives have progressively challenged this model and established sex and gender as important dimensions of rigorous and generalizable research. However, both are multidimensional and intertwined constructs, and their terminology has not always been used consistently across the literature (Ahmed et al., 2026; Maney et al., 2025; Mauvais-Jarvis et al., 2020).

As representation improved, a further challenge became apparent. Including women and men does not in itself ensure that sex-related variation is appropriately considered in study design, analysis or interpretation. Problems in how sex differences are analysed and reported have been documented across the biological sciences (Garcia-Sifuentes and Maney, 2021), while field-specific assessments have revealed similar gaps in areas such as human sleep and circadian physiology (Spitschan et al., 2022).

Attention has therefore moved beyond representation toward deciding which sources of sex-related variation matter for a given research question and how they should be measured. Drawing on the NIH framework of *Consideration, Collection, Characterization and Communication*, Rich-Edwards and Maney outlined how sex can be considered across the research process rather than simply reported as a participant characteristic (Rich-Edwards and Maney, 2023). More recent work has pushed this reasoning further by advocating clearly operationalized, measurable variables rather than treating sex itself as an explanation for the mechanisms underlying an observed difference (Maney et al., 2025). In applied sport and exercise science, ovarian-hormone tracking is similarly framed as purpose-driven and aligned with the research question and setting (Elliott-Sale et al., 2026). Together, these developments mark an important transition from simply including women to asking which sources of variation matter for the question being studied and how they should be characterized.

## When progress raises the threshold

As sex-inclusive standards have become more explicit, the challenge has increasingly shifted from whether sex should be considered to what its rigorous implementation is understood to require. Policy interventions can clearly change practice. For example, at the Canadian Institutes of Health Research, the proportion of grant applications reporting integration of sex increased from 22% to 83%, while integration of gender increased from 12% to 33% between 2011 and 2019 (Haverfield and Tannenbaum, 2021). Yet changes in the published literature have been more uneven. Across nine biological disciplines, studies including both sexes increased from 28% in 2009 to 49% in 2019, while among studies that included both sexes, the proportion analysing data by sex remained statistically unchanged (Woitowich et al., 2020). In sport and exercise science, female accounted for only 34% of more than 12 million participants examined across 5,261 studies published between 2014 and 2020, with 31% of studies including males only compared with 6% including females only (Cowley et al., 2021). A more recent analysis of leading sport and exercise medicine journals suggests that this imbalance persists, with 37% of studies including males only compared with 15% including females only (Cowan et al., 2026). Together, these findings suggest that attention to sex and gender has advanced more rapidly at the policy and funding level than in participant representation and analysis within published research.

Implementation research helps explain why this transition can be difficult. Interviews with 14 principal investigators found that, despite broad support for sex-inclusive research, some interpreted consideration of sex as requiring steroid-hormone measurements, reproductive-cycle tracking or substantially larger sample sizes. These perceptions were accompanied by concerns about cost, time, methodological complexity and limited resources, even though such measures are not intrinsic requirements of the NIH Sex as a biological variable policy (Maney et al., 2023). A recent international survey of 483 pain researchers echoed these concerns, identifying limited expertise, funding, time and human resources among the main barriers to sex- and gender-related research (Finn et al., 2026). The challenge, then, may lie not only in whether researchers consider sex, but in the methodological burden they associate with doing so rigorously.

Seen in this context, our opening example illustrates a broader tension. Current evidence does not establish that increasingly rigorous guidance itself causes women to be excluded, and such a causal claim would go beyond the available literature. It does, however, identify misconceptions and resource constraints through which such an unintended effect could arise. When extensive biological characterization is perceived as a condition of including women (Maney et al., 2023), even when the research question does not require it (Finn et al., 2026; Rich-Edwards and Maney, 2023), standards intended to strengthen research on women may inadvertently raise the threshold for studying them. This is the paradox we need to address.

## Matching rigor to the research question

Resolving this paradox requires separating the inclusion of women from the need for extensive mechanistic characterization. Scientific rigor does not require the same degree of biological characterization for every inferential goal. One study may ask whether an outcome differs by sex; another may ask which variables explain an observed difference. These are related yet distinct questions. Rich-Edwards and Maney distinguish exploratory from confirmatory approaches to sex differences and show that each carries different requirements for study design, statistical power, analysis and data collection (Rich-Edwards and Maney, 2023). In exercise physiology, Sims and Heather similarly distinguish designs intended to compare the sexes from those focused specifically on menstrual cycle or ovarian-hormone effects (Sims and Heather, 2018). Field-specific frameworks have also made the possible depth of biological characterization more explicit. For example, Smith et al. grade menstrual-status characterization and ovarian-hormone control from bronze to silver and gold (Smith et al., 2022). These tiers describe different degrees of methodological control; they should not be interpreted as establishing the highest level of characterization as a universal requirement. Rather, the collected information should depend on the question a study is designed to answer (Rich-Edwards and Maney, 2023). Maney et al. further emphasize that a difference between groups does not itself identify the mechanism that generates it (Maney et al., 2025). The level of biological characterization should therefore follow from the claim a study is designed to support.

Sleep research illustrates why this sequencing matters. A recent systematic review and meta-analysis identified sex-related differences in sleep microarchitecture among adults without insomnia, including higher sigma power, associated with sleep spindles, and delta power, characteristic of deep sleep, in female than in male participants (Walsh et al., 2026). These findings show that sleep physiology differs between sex-classified groups, but do not establish why. Reproductive and hormonal factors may contribute, and studies specifically designed to test their role should measure the relevant variables directly (Maney et al., 2025; Rich-Edwards and Maney, 2023). In applied sport and exercise science, Elliott-Sale et al. similarly emphasize that ovarian-hormone assessment should be purpose-driven (Elliott-Sale et al., 2026). The point is not less rigor, but rigor matched to the question being asked.

## Building the next generation of women’s health research

Improving women’s health will require more than increasingly sophisticated methods. The purpose of methodological rigor is to generate better evidence for prevention, diagnosis, treatment and health outcomes across women’s lives. Mechanistic studies can identify biological processes, while clinical, ecological and population research can show how theses processes interact with social, environmental and structural conditions (Ahmed et al., 2026; Warren et al., 2025). Sex- and gender-related factors are embedded within this wider complexity rather than operating as isolated explanatory domains. Progress will depend on linking evidence across these settings so that mechanistic findings can be tested against real-world patterns and those patterns can, in turn, generate new mechanistic hypotheses (Warren et al., 2025). Clearly operationalized sex- and gender-related variables will be essential to make this evidence interpretable, comparable and cumulative across studies (Finn et al., 2026; Maney et al., 2025).

Making this possible requires more than methodological guidance. Expectations must be matched by the resources needed to meet them, and studies should be judged against the questions they are designed to answer (Rich-Edwards and Maney, 2023). Funders, institutions, journals and research oversight bodies therefore share responsibility for providing the training, expertise, time and infrastructure required to integrate sex and gender rigorously where relevant (Finn et al., 2026). The decade-long experience of the Canadian Institutes of Health Research shows that coordinated policy, training and reviewer support can substantially increase the integration of sex and gender into grant applications (Haverfield and Tannenbaum, 2021). The paradox, then, lies not in rising methodological standards themselves, but in allowing those standards to outpace the capacity to meet them. The next generation of women’s health research will be strengthened not by requiring comprehensive characterisation in every study, but by enabling each study to apply the level of characterisation its question requires while contributing to a broader, connected evidence base. Otherwise, standards intended to strengthen research on women may narrow the questions that can realistically be pursued, leaving important gaps in the knowledge needed to improve women’s health.
